# Novel Molecular Targets for Tumor-Specific Imaging of Epithelial Ovarian Cancer Metastases

**DOI:** 10.3390/cancers12061562

**Published:** 2020-06-12

**Authors:** Lysanne D. A. N. de Muynck, Katja N. Gaarenstroom, Cornelis F. M. Sier, Maurice van Duijvenvoorde, Tjalling Bosse, J. Sven D. Mieog, Cornelis D. de Kroon, Alexander L. Vahrmeijer, Inge T. A. Peters

**Affiliations:** 1 Department of Surgery, Leiden University Medical Center, 2333 ZA Leiden, The Netherlands; l.d.a.n.de_muynck@lumc.nl (L.D.A.N.d.M.); c.f.m.sier@lumc.nl (C.F.M.S.); j.s.d.mieog@lumc.nl (J.S.D.M.); a.l.vahrmeijer@lumc.nl (A.L.V.); 2 Department of Gynecology, Leiden University Medical Center, 2333 ZA Leiden, The Netherlands; k.n.gaarenstroom@lumc.nl (K.N.G.); mauriceduif@live.nl (M.v.D.); c.d.de_kroon@lumc.nl (C.D.d.K.); 3 Department of Pathology, Leiden University Medical Center, 2333 ZA Leiden, The Netherlands; t.bosse@lumc.nl

**Keywords:** epithelial ovarian cancer metastases, surgery, tumor-targeted molecular imaging, near-infrared fluorescence, biomarkers

## Abstract

In epithelial ovarian cancer (EOC), the strongest prognostic factor is the completeness of surgery. Intraoperative molecular imaging that targets cell-surface proteins on tumor cells may guide surgeons to detect metastases otherwise not visible to the naked eye. Previously, we identified 29% more metastatic lesions during cytoreductive surgery using OTL-38, a fluorescent tracer targeting folate receptor-α (FRα). Unfortunately, eleven out of thirteen fluorescent lymph nodes were tumor negative. The current study evaluates the suitability of five biomarkers (EGFR, VEGF-A, L1CAM, integrin αvβ6 and EpCAM) as alternative targets for molecular imaging of EOC metastases and included FRα as a reference. Immunohistochemistry was performed on paraffin-embedded tissue sections of primary ovarian tumors, omental, peritoneal and lymph node metastases from 84 EOC patients. Tumor-negative tissue specimens from these patients were included as controls. EGFR, VEGF-A and L1CAM were highly expressed in tumor-negative tissue, whereas αvβ6 showed heterogeneous expression in metastases. The expression of EpCAM was most comparable to FRα in metastatic lesions and completely absent in the lymph nodes that were false-positively illuminated with OTL-38 in our previous study. Hence, EpCAM seems to be a promising novel target for intraoperative imaging and may contribute to a more reliable detection of true metastatic EOC lesions.

## 1. Introduction

Epithelial ovarian cancer (EOC) is the second most common cause of death among all gynecological malignancies, leading to 125,000 deaths per year worldwide [1]. EOC is commonly referred to as a silent killer, which can be attributed to non-specific symptoms and a lack of effective screening tools. As a result, 75% of patients present with advanced stage disease [2]. The five-year survival rate of advanced stage EOC is approximately 19–29% [3].

The standard treatment for patients with apparent early-stage EOC is a staging procedure consisting of the removal of both ovaries, fallopian tubes, uterus, omentum and lymph nodes and careful the examination of surrounding tissues for the presence of metastatic spread. If no suspicious lesions are detected, biopsies are taken from normal-appearing tissue in predefined areas to determine the extent of disease and the possible need for additional chemotherapy [4]. For patients with advanced-stage disease, in whom complete cytoreduction seems feasible, treatment consists of primary cytoreductive surgery followed by adjuvant platinum-based chemotherapy [5]. Patients with significant co-morbidities, or in whom it is unlikely to reach complete cytoreduction, receive neoadjuvant chemotherapy (NACT) followed by interval cytoreductive surgery. For both early- and advanced-stage disease, the completeness of surgery is the most important prognostic factor for survival [1,6,7,8]. Since the gynecologic oncologist must rely on visual inspection and palpation for detecting metastatic lesions intraoperatively, tumor tissue may remain undetected and potentially lead to undertreatment and subsequent impaired survival. A novel technique which may provide surgical guidance is tumor-targeted molecular imaging.

A leading example of tumor-targeted molecular imaging is near-infrared fluorescence (NIRF) imaging. NIRF imaging can be used intraoperatively to accurately discriminate malignant from benign tissue in real time using fluorescent probes. Ideally, an NIRF probe consists of targeting moieties such as antibodies, peptides or ligands which bind with high affinity to proteins or receptors that are overexpressed on the cell surface of tumor cells but are absent on adjacent normal cells. These targeting moieties are conjugated to a fluorophore which emits light in the near-infrared range (λ = 700–900 nm) [9,10]. The tumor-targeted NIRF probe is intravenously administered to the patient prior to surgery [11], and once the probe has bound to its target, malignant cells will be specifically illuminated and visualized using a tailored optical imaging system. By using NIR wavelengths, a tissue penetration depth of up to 10 mm can be achieved, allowing the detection of malignant cells below the tissue surface [10].

To improve the outcome of cytoreductive surgery by means of tumor-targeted imaging, proteins that are exclusively overexpressed on the cell surface of EOC cells should be identified [12]. Folate receptor alpha (FRα) is overexpressed in 90–95% of epithelial ovarian cancers [13,14] and has therefore been considered a candidate target for NIRF imaging in EOC [7]. Compared to visual inspection alone, the intraoperative use of OTL-38, a folate analogue conjugated to a fluorophore in the NIR range, resulted in the identification of an additional 29% of metastatic peritoneal lesions in patients with advanced-stage EOC [11]. Unfortunately, eleven out of thirteen resected lymph nodes were false positively illuminated.

This study examines the expression of biomarkers to determine their value as novel targets for the NIRF imaging of metastatic EOC lesions with similar accuracy to FRα, but with improved discrimination between tumor-positive and tumor-negative tissue. Recent studies have shown great improvements in understanding the molecular landscape of epithelial ovarian cancer [15]. We focused on biomarkers that exhibit the various hallmarks of cancer, and for which NIRF probes are already clinically available [16,17]: epidermal growth factor receptor (EGFR) [18], vascular endothelial growth factor-A (VEGF-A) [19,20], integrin αvβ6 [21] and epithelial cell adhesion molecule (EpCAM) [9,22]. L1 cell adhesion molecule (L1CAM) was also included, since previous studies have reported overexpression in EOC [23]. FRα was included as a reference marker.

## 2. Results

### 2.1. Patient Selection and Clinicopathological Characteristics

In total, 84 primary tumors, 70 omental, 86 peritoneal, 11 lymph node metastases and 12 tumor samples from diagnostic biopsies (ascites) before NACT derived from 84 patients with EOC, as well as 222 tumor-negative samples (total *n* = 485 samples) from the same patients, were included. Sections from these samples were evaluated by immunohistochemistry to assess expression patterns for EGFR, VEGF-A, L1CAM, EpCAM, integrin αvβ6 and FRα. Tissue samples were derived from 42 patients who underwent either primary cytoreductive surgery (*n* = 297 samples) or 42 patients who underwent interval cytoreductive surgery (*n* = 188 samples). Subgroups were divided based on histological subtype according to the classification model by Shih and Kurman [24,25]: high grade serous carcinoma (HGSC) (*n* = 73) and “other histological subtypes” (*n* = 11), representing low grade serous carcinoma (*n* = 1), endometrioid adenocarcinoma (*n* = 4), clear cell carcinoma (*n* = 2) and mucinous adenocarcinoma (*n* = 4). Patient and tumor characteristics are shown in Table 1.

### 2.2. Evaluation of EGFR, VEGF-A and L1CAM Expression

**Figure 1:**
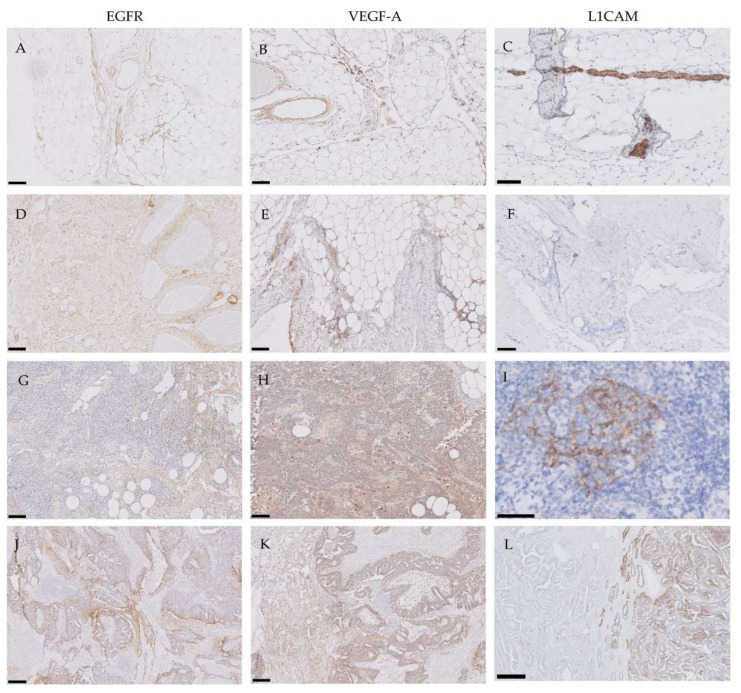
Representative images of the heterogenous expression of epidermal growth factor receptor (EGFR), vascular endothelial growth factor-A (VEGF-A) and L1-cell adhesion molecule (L1CAM) in tumor-negative tissues and primary ovarian tumors. The following structures are shown: tumor-negative omentum (**A**–**C**), tumor-negative peritoneum (**D**–**F**), tumor-negative lymph nodes (**G**–**I**), and primary ovarian tumor (**J**–**L**). Background staining and heterogenous expression in both tumor epithelium and stroma was observed for EGFR and VEGF-A. Scale bars represent 50 µm in I, 100 µm in A–H, 200 µm in J and K and 500 µm in L.

The absence or low expression of molecular targets in adjacent normal tissue is a prerequisite to obtain sufficient contrast using tumor-targeted molecular imaging. However, high background staining was observed in tumor-negative tissues for EGFR, VEGF-A and L1CAM (Figure 1A–I). VEGF-A was expressed by endothelial cells in tumor-negative omentum and peritoneum (Figure 1B, E, respectively), as well as by macrophages in the tumor-negative lymph nodes (Figure 1H). L1CAM was detected on nerves in all tumor-negative tissues and in B cells in lymphatic follicles (Figure 1I).

With respect to the primary ovarian tumors, EGFR, VEGF-A and L1CAM were heterogeneously expressed (Figure 1J–L). EGFR expression was observed in both epithelial cells and surrounding tumor stroma (Figure 1J), and VEGF-A in the cell cytoplasm and surrounding stroma (Figure 1K). Based on their high background staining in tumor-negative tissues and heterogenous expression patterns in primary ovarian tumors, EGFR, VEGF-A and L1CAM were excluded from further analyses.

### 2.3. Expression of EpCAM, αvβ6 and FRα in Tumor-Negative Tissues

**Figure 2:**
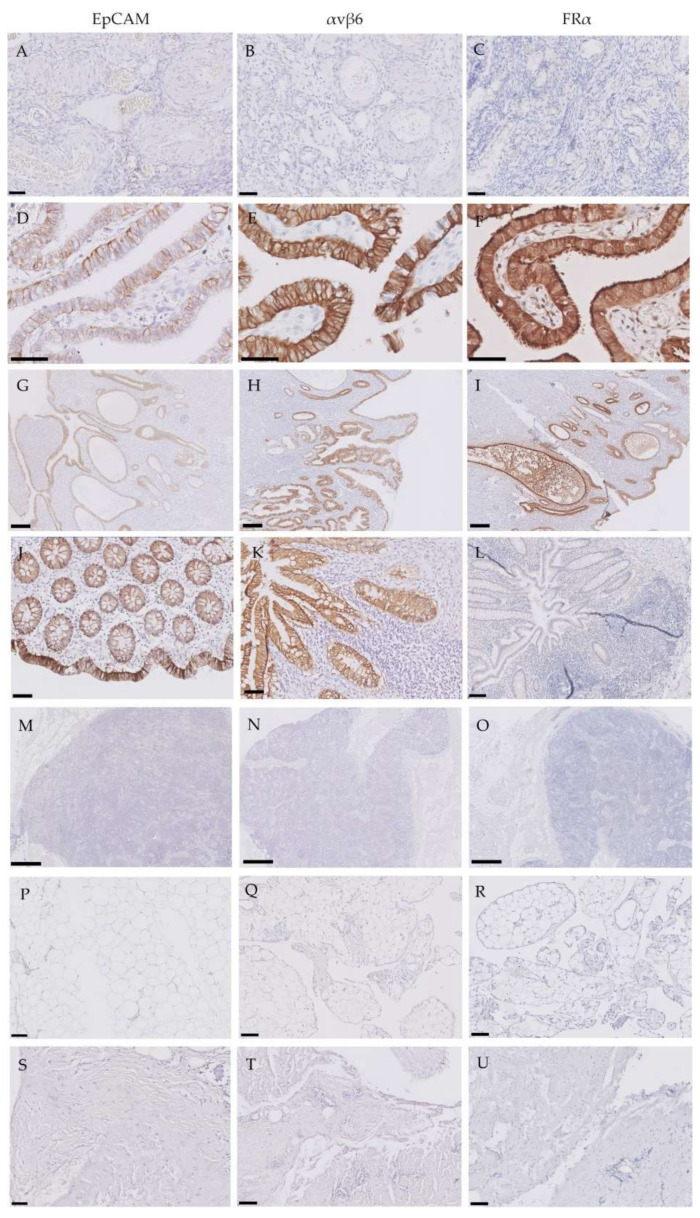
Expression patterns of epithelial cell adhesion molecule (EpCAM), integrin αvβ6 and folate receptor-α (FRα) in tumor-negative tissues. Representative images of immunohistochemically stained tissue samples of tumor-negative ovaries (**A**–**C**), fallopian tubes (**D**–**F**), endometrium (**G**–**I**), intestine (crypt) (**J**–**L**), lymph nodes (**M**–**O**), omentum (**P**–**R**) and peritoneum (**S**–**U**) for EpCAM, αvβ6 and FRα. Scale bars represent 50 µm (**A**–**C**, **K**–**L**), 100 µm (**J**, **M**–**U**), 200 µm (**D**–**F**) and 500 µm (**G**–**I**).

EpCAM, αvβ6 and FRα were detected on the epithelial cells of tumor-negative fallopian tubes (Figure 2D–F), the endometrium (Figure 2G–I) and ovarian inclusion cysts, although EpCAM with lower intensities. Additionally, EpCAM and αvβ6 were expressed with high intensities in the epithelial cells lining the lumen of the small bowel (Figure 2J–K). All other tumor-negative tissue (ovaries, lymph nodes, omentum and peritoneum) showed a complete absence of these markers (Figure 2A–C,L–U).

### 2.4. Expression of EpCAM, αvβ6 and FRα in Lymph Nodes False-Positively Illuminated with OTL-38

**Figure 3:**
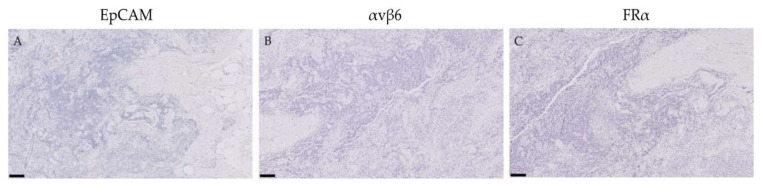
Lymph nodes false-positively illuminated by the fluorescent folate analogue OTL-38 and immunohistochemically stained for EpCAM, αvβ6 and FRα. Representative images of lymph nodes which emitted a false positive signal after the administration of the fluorescent folate analogue OTL-38 in patients with advanced stage EOC, as found in the study by Hoogstins et al. [11]. These lymph nodes (*n* = 11) showed negative expression for EpCAM, αvβ6 and FRα. Scale bars represent 100 µm.

Because the fluorescent folate analogue OTL-38 from the aforementioned study showed false-positive signals in eleven lymph nodes [11], immunohistochemistry was performed on these lymph nodes to determine EpCAM, αvβ6 and FRα expression. All lymph nodes showed a complete absence of these markers (Figure 3).

### 2.5. Expression of EpCAM, αvβ6 and FRα in Primary Ovarian Tumors and Metastases

**Figure 4:**
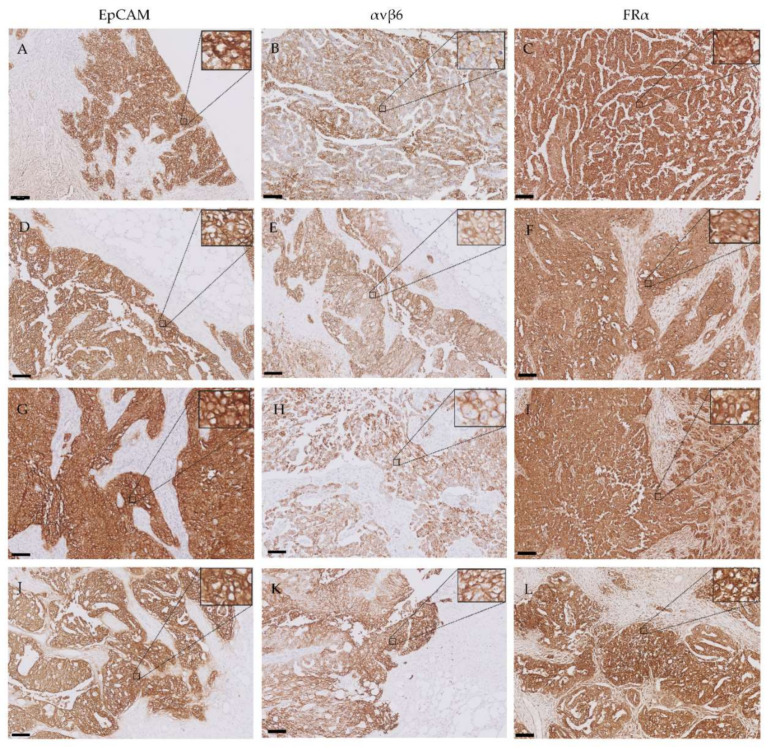
Representative images of primary tumors and their corresponding omental, peritoneal and lymph node metastases immunohistochemically stained for EpCAM, αvβ6 and FRα. The following structures are shown: primary ovarian tumors (**A**–**C**), omental metastases (**D**–**F**), peritoneal metastases (**G**–**I**) and lymph node metastases (**J**–**L**). All images include tumor tissue showing positive expression and adjacent healthy tissue showing no expression. While EpCAM and FRα display high intensity staining in all tumor cells, αvβ6 exhibited more heterogeneous staining intensities in these cells. Scale bars represent 100 µm. Inserts show tumor cells at a higher magnification.

**Figure 5:**
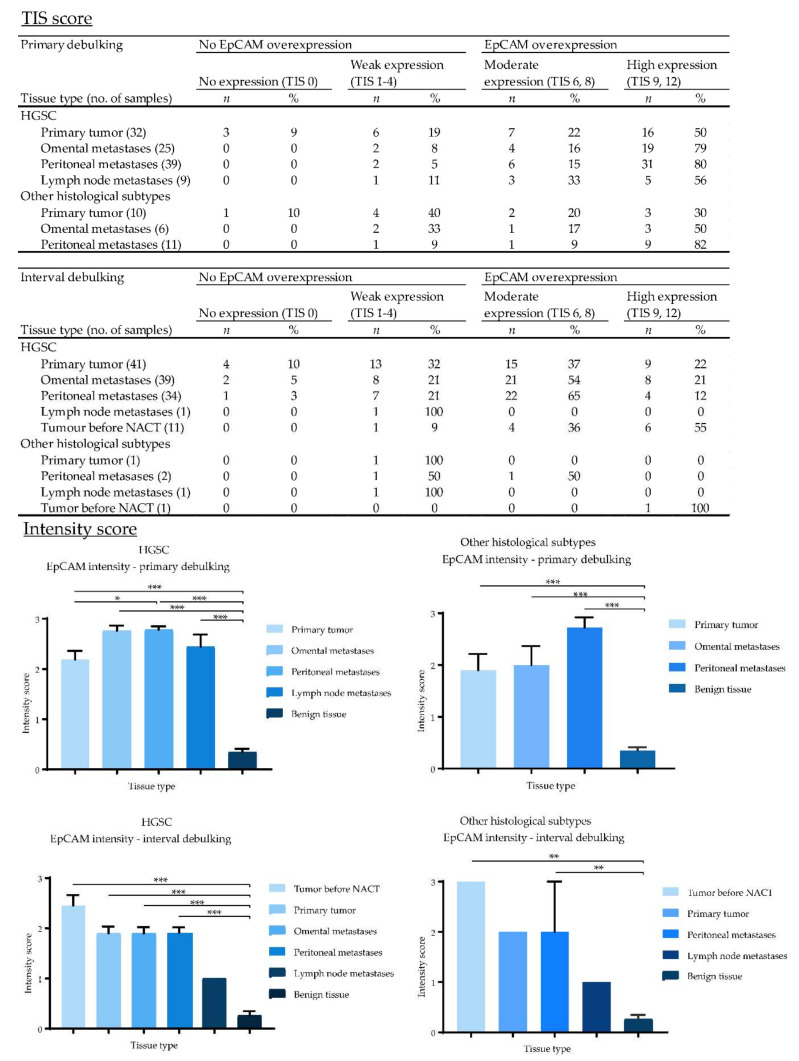
Total immunostaining scores (TIS) and intensity scores of EpCAM for all malignant and benign tissue samples divided by the type of debulking procedure and histological subtype. TIS scores were calculated by multiplying the proportion score (PS), representing the percentage of positively stained tumor cells, with the intensity score (IS) to give one of nine possible values; 0, 1, 2, 3, 4, 6, 8, 9 and 12. Figures with no annotated error bars only represent one sample; for these samples the standard error of the mean (SEM) cannot be provided. (*: *p* < 0.05, **: *p* < 0.01, ***: *p* < 0.001).

**Figure 6:**
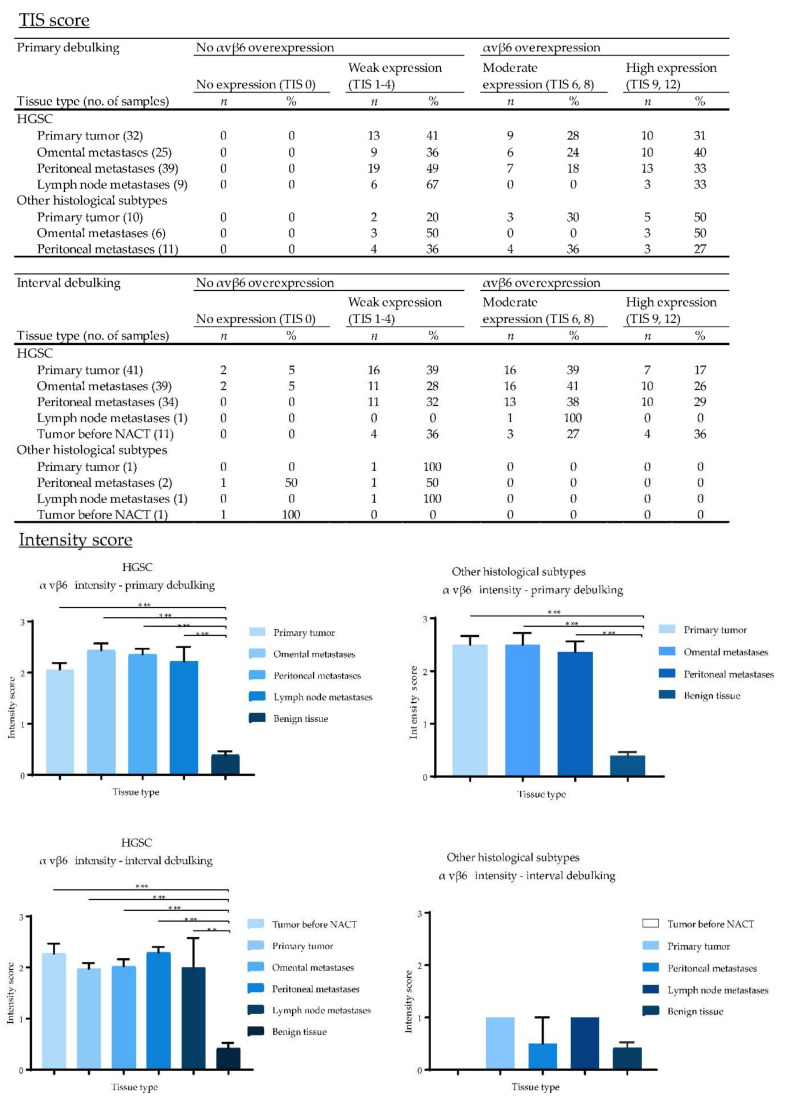
Total immunostaining scores (TIS) and intensity scores of αvβ6 for all malignant and benign tissue samples divided by the type of debulking procedure and histological subtype. TIS scores were calculated by multiplying the proportion score (PS), representing the percentage of positively stained tumor cells, with the intensity score (IS) to give one of nine possible values; 0, 1, 2, 3, 4, 6, 8, 9 and 12. Figures with no annotated error bars only represent one sample; for these samples the standard error of the mean (SEM) cannot be provided. (*: *p* < 0.01, ***: *p* < 0.001).

**Figure 7:**
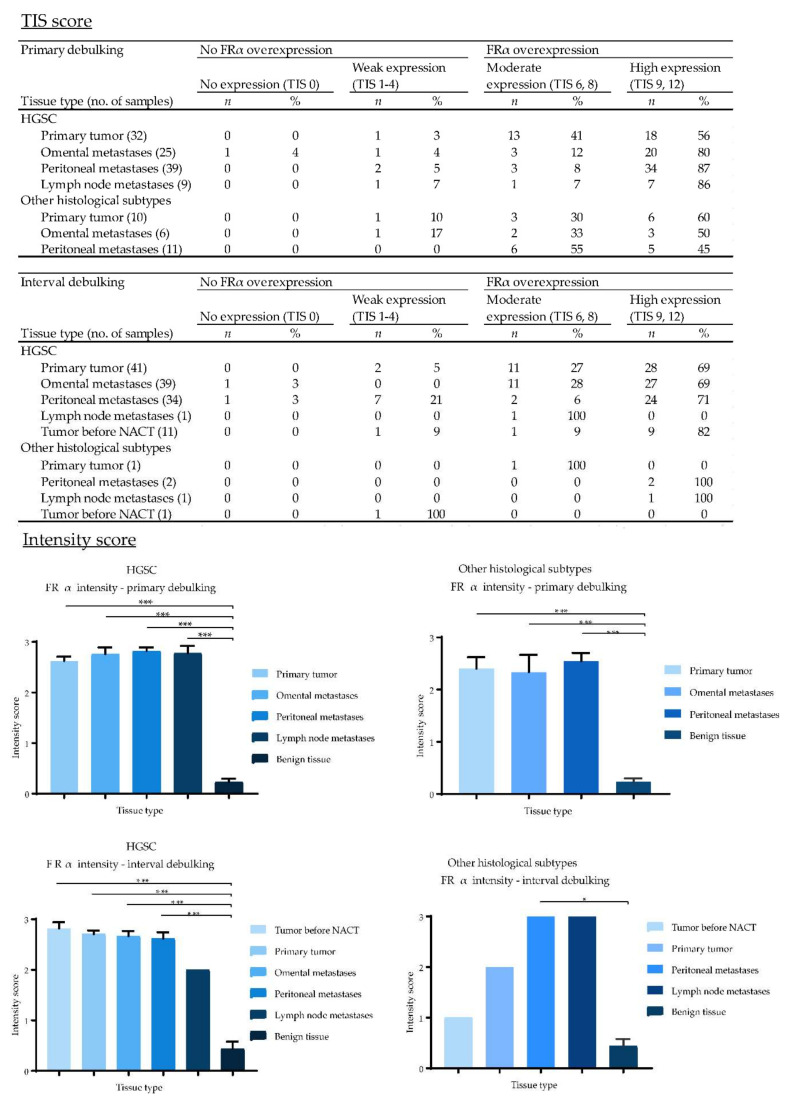
Total immunostaining scores (TIS) and intensity scores of FRα for all malignant and benign tissue samples divided by the type of debulking procedure and histological subtype. TIS scores were calculated by multiplying the proportion score (PS), representing the percentage of positively stained tumor cells, with the intensity score (IS) to give one of nine possible values; 0, 1, 2, 3, 4, 6, 8, 9 and 12. Figures with no annotated error bars only represent one sample; for these samples the standard error of the mean (SEM) cannot be provided. (*: *p* < 0.05, ***: *p* < 0.001).

EpCAM, αvβ6 and FRα were expressed on the cell membranes of metastatic tumor cells (Figure 4). EpCAM showed high homogenous expression patterns, quantified as an average total immunostaining score (TIS) of 9 (Figure 5). An average TIS score of 6 was found for αvβ6, based on its heterogeneous expression patterns, as shown in Figure 4 (middle column) and Figure 6. The expression pattern of EpCAM was similar to the reference marker FRα, as shown in Figure 7. No differences in expression levels were observed across the distinct histological subtypes (Figure 5, Figure 6 and Figure 7).

### 2.6. Lymph Node Detection Accuracy of EpCAM, αvβ6 and FRα

The sensitivity, specificity, positive predictive value (PPV), negative predictive value (NPV) and area under the curve (AUC) were calculated for EpCAM, αvβ6 and FRα based on either “overexpression” or “no overexpression” to determine the potential of correctly identifying tumor-positive and tumor-negative lymph nodes in primary debulking samples (Table 2). αvβ6 showed a lower detection potential than EpCAM and FRα, which showed similar statistics. These statistics could not be calculated for interval debulking samples due to a low sample size of lymph node metastases (*n* = 2).

### 2.7. Biomarker Expression after NACT

To determine whether chemotherapy can affect biomarker expression, tissue samples from both primary and interval cytoreductive surgery were included. When comparing both TIS and intensity scores, there was no statistically significant difference in αvβ6 and FRα expression between primary and interval debulking. However, there was a significant difference in EpCAM expression between both groups in metastases, but not in the primary tumors.

In addition, available diagnostic tumor samples (*n* = 12) before start of NACT from patients in the interval cytoreductive surgery group were immunohistochemically stained for EpCAM, αvβ6 and FRα. Expression patterns before NACT were compared to the remaining vital tumor cells sampled during interval cytoreductive surgery. Although a small decrease was seen in staining intensity for all targets after NACT in the intensity scores bar graphs (Figure 4, Figure 5 and Figure 6), this difference was not statistically significant, suggesting no change in marker expression after being exposed to platinum-based chemotherapeutic agents.

## 3. Discussion

In this study, we demonstrated with immunohistochemistry that EpCAM is a suitable cell-surface protein that can be used as a promising target for tumor-targeted molecular imaging in EOC. Its expression was most comparable to FRα in primary tumors and their corresponding metastases. Furthermore, we showed that both markers were absent in lymph nodes that were previously false-positively illuminated with the fluorescent folate analogue OTL-38. EGFR, VEGF-A and L1CAM appeared to be unsuitable targets, as their high non-specific background staining in tumor-negative tissues could significantly affect the accurate visualization of tumor tissue during surgery. After all, an ideal molecular imaging probe should maximize the target signal in tumor tissue and the minimize background signal in surrounding tumor-negative tissue, leading to an adequate tumor-to-background ratio (TBR). In line with this, the homogenous expression of a biomarker will result in a better demarcation of metastatic deposits than heterogenous expression patterns, making EpCAM and FRα more suitable targets for intraoperative imaging than αvβ6.

Although EpCAM and FRα meet the criteria of a suitable molecular target, high expression was observed in epithelial cells in tumor-negative fallopian tubes and endometrial cells of the uterus. Yet, this will not interfere with surgical performance using intraoperative imaging, as these structures are routinely removed during both surgical staging and cytoreductive surgery. This would only pose a challenge for the application of NIRF-guided surgery in women with early-stage EOC who wish to preserve their fertility. In these patients, the uterus and contralateral ovary and fallopian tube will be left in situ in case of normal appearance [4]. As a result, the epithelial cells of the contralateral fallopian tube and endometrium might be illuminated, making it impossible to determine whether the tubes or uterus harbor any malignant cells. However, previous research within our group showed that an intraoperative fluorescence signal in tumor-negative structures expressing FRα could be clearly distinguished from the signal measured in tumor deposits [11].

Similarly, EpCAM expression was observed in epithelial cells lining the intestinal lumen. Since an NIRF probe has a tissue penetration depth of up to 1 cm, these cells may be detected by a probe targeting EpCAM and result in background fluorescence. On the contrary, it is possible that EOC metastases located on the serosal surface of the small bowel can still be adequately discriminated. After all, it has been shown that there are four times as many EpCAM molecules expressed on a malignant cell compared to a normal cell, and while this may not be observed by immunohistochemistry, a difference in fluorescence intensity could be perceived [23]. Hence, there may be a difference between protein quantification based on immunohistochemical analyses and real-life in vivo scenarios. Further intraoperative validation using NIRF imaging targeting EpCAM would be a suggested future step to determine whether EOC metastases located on the serosa of the small bowel can be adequately distinguished from normal epithelial cells lining the intestinal lumen.

Strikingly, no expression of EpCAM, αvβ6 or FRα was observed in the lymph nodes that were previously false-positively illuminated with OTL-38. It has been suggested that the false positive signal detected in the lymph nodes was likely due to the fact that OTL-38 not only binds to FRα, but also FRβ, which is expressed by tumor-associated macrophages (TAMs) present in the lymph nodes. The presence of FRβ in the previous false positive lymph nodes was confirmed via immunostaining [11]. Since, at present, there is no clinical probe available which solely targets FRα, there is an ongoing need for more specific targets for the molecular imaging of metastatic EOC lesions which can be used to optimize surgical procedures. EpCAM was able to correctly identify all primary debulking tumor-negative and tumor-positive lymph nodes, with a sensitivity, specificity, PPV, NPV and AUC of 88%, 100%, 100%, 94% and 0.97 (95% CI 0.90–1.00), respectively. These results further highlight the suitability of EpCAM as a molecular target, as its targeting specificity would decrease the chance of non-specific uptake in tumor-negative tissue. Due to a low sample size, these statistics could not be calculated for interval debulking samples.

Increases in EpCAM expression have been correlated to cancer development and progression and increased proliferation [9,26,27], which is also evident in our results. An increase in EpCAM expression was detected in metastatic tissue compared to primary ovarian tumors, which could eliminate the need to firstly immunohistochemically analyze primary tumors for target identification prior to surgery. In addition, to determine the effect of exposure to NACT on target expression, diagnostic tumor samples before NACT, if applicable, were included from patients who underwent interval cytoreductive surgery. Although the expression of EpCAM was not significantly altered following NACT in this analysis, it should be noted that the sample size was relatively low, as only twelve diagnostic samples were included. On the contrary, when examining this effect in a larger cohort by comparing the expression of EpCAM in malignant tissues from patients who underwent primary cytoreductive surgery to those derived from patients who underwent interval cytoreductive surgery, a statistically significant decrease in EpCAM expression was observed in patients with HGSC tumors. Nevertheless, since EpCAM expression levels remained high following NACT, this decrease would not be clinically relevant, as our results showed there would still be a sufficient fluorescence signal to allow for the accurate detection of metastatic lesions in HGSC patients undergoing interval cytoreductive surgery. Whether this will also apply to patients diagnosed with histological subtypes other than HGSC cannot be stated with certainty, as the sample size was too low to draw firm conclusions.

Beside the intraoperative use, tumor-targeted molecular imaging may also be used pre-operatively to determine tumor load and surgical resectability. The pre-operative estimation of surgical resectability in EOC is currently based on computed tomography (CT) scans. However, a CT scan may underestimate the extent of disease. As a result, a significant proportion of patients with advanced stage disease will undergo a futile laparotomy [28,29,30]. Better selection of patients in whom complete cytoreduction at primary cytoreductive surgery can be achieved will reduce morbidity. In a randomized study in the Netherlands, it was found that a diagnostic laparoscopy prior to cytoreductive surgery reduced the number of futile laparotomies (i.e., residual disease > 1 cm) from 39% to 10% [31]. However, a diagnostic laparoscopy is an invasive procedure and has its limitations, such as proper inspection of the whole mesentery and serosa of the bowel [28]. Currently, it is being investigated whether diffusion-weighted magnetic resonance imaging (DW-MRI) can be used to predict surgical resectability, as DW-MRI has a very high sensitivity to detect small volume malignant disease (www.clinicaltrials.gov, NCT03399344). Another promising technique to preoperatively assess metastatic tumor load in patients with EOC might be a pre-operative tumor-targeted positron emission tomography (PET) scan using tumor-specific radioactive tracers. To use EpCAM as a target for tumor-specific PET imaging, antibodies or peptides against EpCAM can be conjugated to a radioactive isotope. A combination of tumor-specific PET and NIRF imaging could further improve the detection of metastatic lesions.

## 4. Materials and Methods

### 4.1. Patient and Tissue Selection

Medical records and tissue specimens were retrospectively reviewed from patients diagnosed with advanced stage EOC (FIGO IIb-IV [2]) who underwent cytoreductive surgery between 2011–2017 at the Leiden University Medical Center (LUMC), The Netherlands. Hematoxylin and eosin stained tissue sections retrieved from the archives of the Department of Pathology were reviewed by a pathologist specializing in gynecologic oncology (TB) to determine the histological subtype and to select the most suitable tissue specimens for immunohistochemical analysis. This resulted in the inclusion of tissue from 42 patients who underwent primary cytoreductive surgery and 42 patients who underwent interval cytoreductive surgery following NACT, of whom a large number of metastatic tissue samples was available. Tissue samples included primary tumors (*n* = 84), their corresponding omental, peritoneal and lymph node metastases (*n* = 167) and tumor tissue from diagnostic biopsies before NACT, if applicable (*n* = 12). Tumor-negative tissue specimens in the primary debulking group originated from patients who underwent a staging procedure because of suspected early-stage EOC. Tumor-negative tissue specimens in the interval debulking group originated from biopsies taken during interval debulking procedures and were therefore subject to NACT. To determine expression in tumor-negative tissue from surrounding structures, samples from the ovaries, fallopian tubes, uterus, omentum, pouch of Douglas, bladder peritoneum, pelvic wall, paracolic gutter, diaphragm, intestine/appendix and lymph nodes were included from all patients (*n* = 222). Subjects who were alive at the time of the study gave their informed consent for the use of their tissue. This study was conducted in accordance with the Declaration of Helsinki and approved by the Ethics Committee (protocol number B17.025).

Because previous research has indicated heterogenous expression levels of EGFR, VEGF-A and L1CAM in EOC [19,20,32], a pilot staining was primarily conducted. Samples included a selection of primary tumors (*n* = 5) and tumor-negative peritoneal (*n* = 3), omental (*n* = 3) and lymph node (*n* = 3) tissue.

Additionally, considering the aforementioned false-positivity in lymph nodes reported in a translational study with an FRα targeting probe (OTL-38) by our group [11], these false positive lymph node specimens (*n* = 11) were immunohistochemically stained for the most promising targets.

### 4.2. Immunohistochemistry

Whole formalin-fixed paraffin-embedded (FFPE) tissue blocks were cut into sections of 4 µm thickness. After the deparaffinization of these sections in xylene and rehydration in a stepwise series of alcohol solutions, endogenous peroxidase activity was blocked with 0.3% hydrogen peroxide for 20 min. Deparaffined slides were placed in a 37 °C water bath and incubated with 0.125% trypsin for 30 min to unmask EpCAM epitopes, and with 0.4% pepsin and 1N HCl for 20 min to unmask αvβ6 and EGFR epitopes. VEGF-A epitopes were unmasked by heat induction at 95 °C using citrate buffer (pH 6.0, Dako, Glostrup, Denmark) in a PT-Link module (Agilent, Santa Clara, CA, USA). Antigen retrieval for L1CAM was performed by a 30 min incubation in Tris-EDTA (pH 9.0, Dako, Glostrup, Denmark) pre-heated to 90–100 °C. The slides were then incubated overnight in a humidified chamber at room temperature with previously determined dilutions of primary monoclonal antibodies against EpCAM (0.25 µg/mL), αvβ6 (0.5 µg/mL), EGFR (2.86 µg/mL), VEGF-A (0.25 µg/mL) and L1CAM (1 µg/mL). The slides were then washed in PBS and incubated for 30 min at room temperature with a horseradish peroxidase (HRP)-labelled secondary antibody (anti-mouse or anti-rabbit Envision, Dako). Staining for FRα was performed using an immunohistochemical assay kit according to the manufacturer’s instructions (BioCare Medical, Pacheco, CA, USA). After being rinsed in PBS, the immunoreactions were visualized using DAB substrate buffer (Dako) for 10 min and counterstained using Mayer’s hematoxylin for 15–30 s. After dehydration at 37 °C, the stained slides were mounted with pertex (Leica Microsystems, Wetzlar, Germany). All antibodies and reagents used for immunohistochemical staining can be found in Appendix A.

### 4.3. Evaluation of Immunoreactivity

All immunohistochemically stained slides were scanned using the Ultra-Fast Scanner 1.6 RA (Philips, Eindhoven, The Netherlands). Staining in malignant tissues was evaluated based on membranous expression. Positive expression was scored independently by two blinded observers (L.M. and M.D.), of which a final agreement score was determined. Samples with no agreement were resolved by consensus. The score comprised the total amount of positively stained tumor cells as a percentage of total tumor tissue (proportion score (PS): 0 ≤ 9%, 1 = 10–25%, 2 = 26–50%, 3 = 51–75%, 4 ≥ 76%), and the staining intensity (intensity score (IS): 0 = none, 1 = weak, 2 = moderate, 3 = strong). Examples of staining intensities can be seen in Appendix B. These scores were then multiplied (PS x IS) to give a final total immunostaining score (TIS) of one of nine possible values: 0 = no expression, 1, 2, 3, 4 = weak expression, 6, 8 = moderate expression, 9, 12 = strong expression. Tumor-negative tissue sample scores were based solely on staining intensity as 0 = none, 1 = weak, 2 = moderate, 3 = strong, as the percentage of positively stained tumor cells is not applicable.

### 4.4. Statistical Analysis

All statistical analyses were performed using SPSS software version 23 (SPSS, IBM Corporation, Somer, NY, USA). Agreement scores were calculated using Cohen’s kappa. A one-way ANOVA with a post-hoc Bonferroni correction was applied to calculate mean percentage staining and the difference in staining between tissues. Results were considered statistically significant if *p* < 0.05. The sensitivity, specificity, positive predictive value (PPV), negative predictive value (NPV) and area under the curve (AUC) were calculated for all tumor-negative and tumor-positive lymph nodes for EpCAM, αvβ6 and FRα. Calculations were based on the TIS scores and were classified as “overexpression” (TIS 6–12) and “no overexpression” (TIS 1–4). Tumor-negative lymph nodes with no observed expression were classified as “no overexpression”. Sensitivity was calculated by dividing the lymph nodes with true overexpression by the total number of tumor-positive lymph nodes. Specificity was calculated by dividing the lymph nodes with true no overexpression by the total number of tumor-negative lymph nodes. PPV was calculated by dividing the lymph nodes with true overexpression by the total number of lymph nodes with overexpression, and NPV was calculated by dividing the lymph nodes with true no overexpression by the total number of lymph nodes with no overexpression. The AUC was calculated using SPSS. Further graphical representations of staining intensities were created using GraphPad Prism 7 (GraphPad, Software, Inc, La Jolla, CA, USA). Image representations of immunohistochemical stainings were created using Adobe Illustrator CC 2018 (Adobe Systems Inc., San Jose, CA, USA).

## 5. Conclusions

In conclusion, our study showed that EpCAM is a potential alternative target for FRα for tumor-specific intra-operative molecular imaging for EOC. Since a high and homogenous expression was detected in primary tumors and metastases, and no false positivity in lymph nodes was observed, EpCAM may contribute to a more reliable detection of metastatic EOC lesions. Further research is needed to determine whether guidance by tumor-targeted molecular imaging will more often lead to the completeness of surgery.

## Figures and Tables

**Table 1:** Clinicopathological characteristics of 84 epithelial ovarian cancer patients.

Clinicopathological Characteristics	Primary Debulking (42)	Interval Debulking (42)
Age in years, median (range)	65 (29–83)	67 (30–88)
FIGO ^1^ stage [2]		
IIB	3	0
IIIA	1	0
IIIB	6	1
IIIC	31	25
IV	1	16
Histological subtype		
HGSC ^2^	32	41
Other ^3^	10	1
LGSC ^4^	1	0
Endometrioid adenocarcinoma	4	0
Clear cell carcinoma	1	1
Mucinous adenocarcinoma	4	0
BRCA carrier status		
Positive, BRCA1	5	4
Positive, BRCA2	1	1
Negative	16	20
Unknown	20	17

^1^ FIGO: International Federation of Gynecology and Obstetrics [2] ^2^ HGSC: high grade serous carcinoma ^3^ “Other” includes LGSC, endometrioid adenocarcinoma, clear cell carcinoma and mucinous adenocarcinoma. ^4^ LGSC: low grade serous carcinoma.

**Table 2:** Overview of lymph node detection potential of EpCAM, αvβ6 and FRα.

Primary Debulking					
Biomarker	Sensitivity	Specificity	PPV ^1^	NPV ^2^	AUC ^3^ (95% CI)
EpCAM	88%	100%	100%	94%	0.97 (0.90 to 1.00)
αvβ6	33%	100%	100%	71%	0.86 (0.70 to 1.00)
FRα	88%	100%	100%	94%	0.97 (0.90 to 1.00)

^1^ PPV: positive predictive value; ^2^ NPV: negative predictive value; ^3^ AUC: Area under the curve.

**Figure A1:**
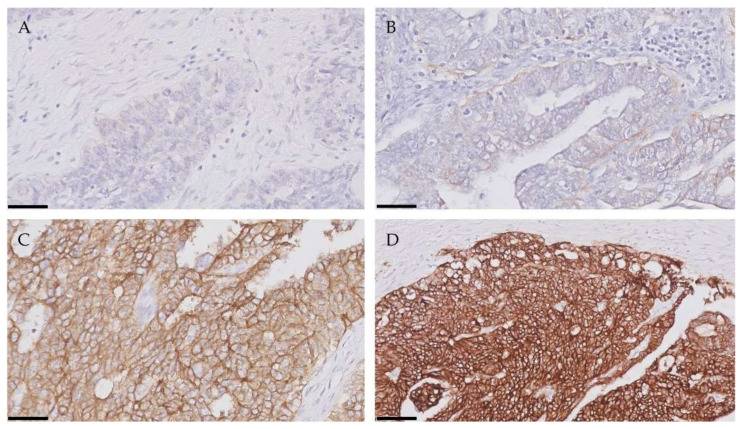
Evaluation of immunoreactivity in primary ovarian tumors: intensity scores. The following intensities are shown: intensity 0 (**A**), intensity 1 (**B**), intensity 2 (**C**), and intensity 3 (**D**). Scale bars represent 50 µm.

## Appendix A

**Table A1:** Antibodies and reagents used for immunohistochemical stainings.

Antibody	Clone Number	Species	Monoclonal/Polyclonal	Stock Concentration	Dilution	Company
*Primary antibodies*						
Anti-EGFR	E30	Mouse	Monoclonal	286 µg/mL	1:100	Dako, Glostrup, Denmark
Anti-VEGF-A	RB9031	Rabbit	Monoclonal	200 µg/mL	1:800	Thermo Fisher Scientific, Waltham, MA, USA
Anti-L1CAM	14.10	Mouse	Monoclonal	0.5 mg/mL	1:500	BioLegend, San Diego, CA, USA
Anti-EpCAM	323/A3	Mouse	Monoclonal	0.4 mg/mL	1:1600	Department of Pathology, LUMC, The Netherlands
Anti-αvβ6	6.2A1	Mouse	Monoclonal	50 µg/mL	1:100	Biogen Idec MA Inc., Cambridge, MA, USA
Anti-FR-α	BRI 4006K AA (kit)	Mouse	Monoclonal	N/A	Ready-to-use	Biocare Medical, Pacheco, CA, USA
